# *CDK4 *IVS4-nt40 AA genotype and obesity-associated tumors/cancer in Italians – a case-control study

**DOI:** 10.1186/1756-9966-28-42

**Published:** 2009-03-28

**Authors:** Ramachandran Meenakshisundaram, Claudia Gragnoli

**Affiliations:** 1 Department of Medicine and Cellular & Molecular Physiology, Milton S. Hershey Medical Center, Hershey, PA, USA; 2 Penn State University College of Medicine, Hershey, PA, USA; 3 Sbarro Institute for Cancer Research & Molecular Medicine, Center for Biotechnology, Temple University's College of Science & Technology, Philadelphia, PA, USA; 4 Department of Biology, Temple University's College of Science & Technology, Philadelphia, PA, USA; 5 Molecular Biology Laboratory, Bios Biotech Multi-Diagnostic Health Center, Rome, Italy

## Abstract

**Background:**

Cell cycle checkpoint regulation is crucial for prevention of tumor in mammalian cells. Cyclin-dependant kinase 4 (CDK4) is important in cell cycle regulation, as it controls the G1-S phase of the cell cycle. CDK4 has potential mitogenic properties through phosphorylation of target proteins. We aimed at identifying a role of *CDK4 *IVS4-nt40 G→A gene variant in benign and/or malignant tumors and in obesity-associated benign and/or malignant tumors in an Italian adult subject dataset.

**Methods:**

We recruited 263 unrelated Italian subjects: 106 subjects had at least one benign tumor and 46 subjects had at least one malignant tumor, while 116 subjects had at least two tumors and/or cancers. We collected BMI data for 90% of them: 186 subjects had a BMI≥30 Kg/m^2 ^and 52 subjects had a BMI ≥ 30 Kg/m^2^. We performed statistical power calculations in our datasets. DNA samples were directly sequenced with specific primers for the *CDK4 *IVS4-nt40 G→A variant. Genotype association tests with disease were performed.

**Results:**

In our study, no significant association of the *CDK4 *IVS4-nt40 AA genotype with cancer and/or tumors/cancer are/is detected. However, the *CDK4 *IVS4-nt40 AA genotype is significantly associated with cancer and tumors/cancer in obese patients.

**Conclusion:**

This finding is interesting since obesity is a risk factor for tumors and cancer. This study should prompt further work aiming at establishing the role of *CDK4 *in contributing to tumor/cancer genetic risk predisposition, as well as its role as a potentially effective therapeutic target gene for obesity-associated tumor/cancer management.

## Introduction

Cell cycle checkpoint functions regulate cell cycle progression and proliferation. Defects of cell cycle control are one among hallmarks of tumor development and may have relevance in tumor predisposition [1]. Cyclin-dependant kinase 4 (*CDK4*) is an important gene for cell cycle regulation, as it determines the number of cells entering the G1 phase cell cycle [2]. It is located on chromosome 12q14 and the protein encoded within this gene is a member of Ser/Thr protein kinase family. CDK4 has mitogenic [2] through phosphorylation of target proteins [4]. The chromosome 12q12-q14 region has been shown by a genome scan to be in linkage to bladder cancer [5], as well as to obesity-associated type 2 diabetes genes [6]. Previous studies have reported differential *CDK4 *expression in tumors such as gliosarcoma, mantle cell lymphoma and squamous cell carcinoma [7-9]. However, no study has up to date investigated the *CDK4 *variant in the human genome of cancer patients to prove their potential role in oncogenic pathogenesis.

This study was carried out to find out whether there is any association of *CDK4 *IVS4-nt40 G→A SNP with cancer and/or tumors/cancer as well as with obesity-associated cancer and/or tumors/cancer in the Italian population.

## Materials and methods

We recruited from Italy a total of 263 unrelated adult subjects from the general population. We carried out the study with the written informed consent from each subject and with the approval from the Institutional Review Board, in accordance with the Helsinki Declaration guidelines. We collected clinical information on the presence or absence of tumors and/or cancer on the total 263 subjects. Among 263 subjects, 152 subjects (58%) presented with either benign and/or malignant tumors: among these, 106 subjects had at least one benign tumor and 46 subjects had at least one malignant tumor, while 116 subjects had at least two tumors and/or cancer. The various tumor and cancer types are described in Table 1.

**Table 1:** Number of tumors/cancers types

**Site**	**Tumor**	**Cancer**
Skin	1	6

Oral cavity	1	1

RT including lungs	2	2

GIT	8	8

Hormonal	67	22

Thyroid	29	1

Hematological	1	5

Brain	3	1

Endocrine	2	0

RT = Respiratory tract, GIT = Gastrointestinal tract (liver, colon and pancreas), Hormonal-dependent = Breast, Ovary, Uterus, Prostate

In the subject group, we collected BMI data for 90% of subjects: 186 subjects had a BMI less than 30 Kg/m^2 ^and 52 subjects had a BMI≥30 Kg/m^2^, thus the latter met the definition for obesity.

DNA samples were directly sequenced by PCR and automated fluorescence sequencer with specific primers for the *CDK4 *IVS4-nt40 G→A single nucleotide polymorphism (SNP).

True detectable odds ratios (ORs) for genotype association tests were calculated in our datasets with statistical power at least 60%, type 1 error probability of 0.05, and given, in the general Italian population, a cancer prevalence of 2.7% [10] and, in the obese Italian population, of 3.2% [11] (Table 2).

**Table 2:** Statistical power calculated for genotype association test in each case-control dataset with α = 0.05

**Subject groups**	**Power**	**Detectable OR**
46 cases and 204 control subjects	65%	4.435

152 cases and 111 control subjects	65%	4.400

10 cases and 178 control subjects	65%	7.975

23 cases and 89 control subjects	60%	5.725

OR = odds ratio

We tested the *CDK4 *IVS4-nt40 G→A SNP alleles for departure from Hardy-Weinberg equilibrium (HWE) in our cases (positive cancer and/or tumors/cancer) and control subjects (with no cancer and no tumors/cancer) groups, separately, by using Chi-Square test statistics.

With the Mantel-Haenszel algorithm, we tested the *CDK4 *IVS4-nt40 G→A genotype variant for association with cancer and with tumors/cancer against control subjects with no cancer and no tumors/cancer, respectively. We further tested the *CDK4 *IVS4-nt40 G→A at genotype level for association with obesity-associated cancer and with obesity-associated tumors/cancer against non-obese control subjects with no cancer and no tumors/cancer, respectively.

We also perfomed an association test for non-obese cancer and tumors/cancer cases.

## Results

All alleles tested in each group of the four datasets were not in departure from HWE.

We did not identify in our dataset any significant and valid association of the *CDK4 *IVS4-nt40 G→A genotype variant with either cancer or tumors/cancer against control subjects with no cancer and no tumors/cancer, respectively (Table 3, 4). However, our dataset may not be able to detect any risk variant with a modest effect contributing to cancer and/or tumors/cancer.

**Table 3:** CDK4 IVS4-nt40G→A genotype association with cancer

**Genotype**	**46 cancer**		**204 No cancer**		**X^2^**	**2-t P**	**OR**	**95% C.I.**
					
	+	-	+	-				
AA	7	39	14	190	3.405	0.060	2.44	0.83 – 7.00

AG	20	26	76	128	0.615	0.433	1.30	0.64 – 2.60

GG	19	27	114	90	3.204	0.073	0.56	0.28 – 1.11

X^2 ^= Chi-Square, 2-t P = 2-tailed p-value, OR = odds ratio, C.I. = confidence interval

**Table 4:** CDK4 IVS4-nt40G→A genotype association with tumor/cancer

**Genotype**	**152 Tumor/cancer**		**111 No tumor/cancer**		**X^2^**	**2-t P**	**OR**	**95% C.I.**
					
	+	-	+	-				
AA	19	133	6	105	3.754	0.053	2.50	0.90 – 7.28

AG	57	95	52	59	2.309	0.129	0.68	0.40 – 1.15

GG	76	76	53	58	0.130	0.718	1.09	0.65 – 1.84

X^2 ^= Chi-Square, 2-t P = 2-tailed p-value, OR = odds ratio, C.I. = confidence interval

In the subset of the obesity-associated tumor/cancer analysis, we identified a significant association of the *CDK4 *IVS4-nt40 AA genotype with BMI ≥ 30 and cancer (P = 0.002, Table 5), and with BMI ≥ 30 and tumors/cancer (P = 0.007, Table 6). We had in our datasets of genotype association tests with the obesity-associated cancer and obesity-associated tumors/cancer at least 60% power to detect the identified risk ORs identified (Table 2). The analysis performed to exclude association of the *CDK4 *IVS4-nt40 AA genotype with the subset of non-obese cancer and tumors/cancer was not significant (data not shown).

**Table 5:** CDK4 IVS4-nt40G→A genotype association with cancer and BMI ≥ 30

**Genotype**	**10 Cancer and BMI ≥ 30**		**178 No cancer and BMI<30**		**X^2^**	**2-t P**	**OR**	**95% C.I.**
					
	+	-	+	-				
AA	3	7	9	169	9.858	0.002	8.05	1.37 – 44.21

AG	2	8	66	112	1.196	0.274	0.42	0.06 – 2.25

GG	5	5	103	75	0.240	0.624	0.73	0.17 – 3.03

BMI = body mass index, X^2 ^= Chi-Square, 2-t P = 2-tailed p-value, OR = odds ratio, C.I. = confidence interval

**Table 6:** CDK4 IVS4-nt40G→A genotype association with tumor/cancer and BMI≥30

**Genotype**	**23 Tumor/cancer and BMI ≥ 30**		**89 No tumor/cancer and BMI<30**		**X^2^**	**2-t P**	**OR**	**95% C.I.**
					
	+	-	+	-				
AA	5	18	4	85	7.355	0.007	5.90	1.21 – 29.82

AG	8	15	35	54	0.159	0.690	0.82	0.28 – 2.35

GG	10	13	50	39	1.185	0.276	0.60	0.22 – 1.66

BMI = body mass index, X^2 ^= Chi-Square, 2-t P = 2-tailed p-value, OR = odds ratio, C.I. = confidence interval

## Discussion

CDK4 is the catalytic subunit of the cyclin D-CDK holoenzyme. The kinase activity of this complex is induced in response to extracellular signals, including growth factors, and it translates signals from the extracellular environment into cell cycle activation. The *CDK4 *gene lies in a chromosomal region of interest for cancer predisposition [4] and for obesity-associated T2D genes [5]. It is known to be involved in cell cycle regulation, and represents a strong candidate gene for tumor and/or cancer genetic predisposition [6-8]. Although the effect size of any potential gene risk variant in any tumor/cancer is not predictable until is tested, we can deduct from the present study that the *CDK4 *IVS4-nt40 AA genotype does not independently and significantly contribute as a major significant risk variant to tumors/cancer in our Italian dataset. If there is any *CDK*4 variant risk effect in tumor and/or cancer predisposition, it is likely too modest to be detected in the current dataset. It is possible, however, that other *CDK4 *gene variants may potentially contribute to tumor/cancer risk predisposition as well as that any potential *CDK4 *variant association may be detected by using a larger dataset.

On the contrary, it should also be considered that the tumor/cancer risk predisposition may be linked to the obesity-factor. In fact, in our study, obese patients (BMI ≥ 30) with *CDK4 *IVS4-nt40AA genotype have a significant increased risk for cancer and tumors/cancer, in both datasets tested. As we excluded any association of the *CDK4 *IVS4-nt40 AA genotype with the subset of non-obese cancer and tumor/cancer cases, we were able to further confirm the validity of the identified association with the obese-associated cancer and tumor/cancer cases.

Several studies report that obesity increases tumor/cancer incidence [10-12]. From our study, we may conclude that *CDK4 *IVS4-nt40 AA genotype plays a role in obesity-associated tumor/cancer risk predisposition. However, more studies are warranted to establish the role of other *CDK4 *variants in tumor-cancer predisposition [4]. As obesity is a preventable associated factor in several tumor and/or cancer types [10-12], both lifestyle modification and genetic screening for obesity-associated tumor/cancer gene risk variants should be implemented to prevent tumors and cancer in patients.

## Competing interests

The authors declare that they have no competing interests.

## Authors' contributions

CG participated in study design, DNA amplification, sequence reading, project coordination and manuscript drafting and revising. RM carried out the statistical analysis, reference collection, and manuscript drafting. All authors have read and approved the manuscript.
